# Sex and age differences in glia and myelin in nonhuman primate and human spinal cords: implications for pathology

**DOI:** 10.1038/s41420-025-02425-9

**Published:** 2025-04-02

**Authors:** Gaëtan Poulen, Nacéra Douich, Chloé M. Gazard, Nadine Mestre-Francés, Maïda Cardoso, Luc Bauchet, Florence Vachiery-Lahaye, Nicolas Lonjon, Yannick N. Gerber, Florence E. Perrin

**Affiliations:** 1 MMDN, Univ. Montpellier, EPHE, INSERM, Montpellier, France; 2 Department of Neurosurgery, CHU Montpellier, France; 3 PSL Research University, Paris, France; 4 University of Montpellier, plateforme BNIF, Montpellier, France; 5 INSERM U1191, Institute of Functional Genomics, University of Montpellier, Montpellier, France; 6 Department of coordination hospitalière des dons pour la greffe, CHU Montpellier, France; 7 Institut Universitaire de France (IUF), Paris, France

**Keywords:** Glial biology, Spine regulation and structure

## Abstract

In a healthy central nervous system, glial cells are influenced by genetic, epigenetic, age, and sex factors. Aging typically causes astrocytes and microglia to undergo changes that reduce their neuroprotective functions and increase harmful activities. Additionally, sex-related differences in glial and myelin functions may impact neurological disorders. Despite this, few studies have investigated glial cells in primates, with most focusing on the brain. This study aims to explore whether glial cells and myelin exhibit age- and sex-related differences in the spinal cord of nonhuman primates and humans. We used immunohistochemistry and myelin staining to analyze healthy spinal cord samples from midlife and aged individuals of both sexes, focusing on *Microcebus murinus* (a small nonhuman primate) and humans. Primate spinal cords show distinct variations in glial markers and myelin characteristics related to sex and age, with differences varying between species. Notably, GFAP expression is sex-dependent in both primate species. We also observed greater differences in the expression of microglial markers than other glial markers. Overall, we found the opposite pattern for the g-ratio and oligodendrocytic marker between species. These findings suggest that glial cells may play a critical role in age- and sex-related differences in the prevalence and progression of spinal cord diseases.

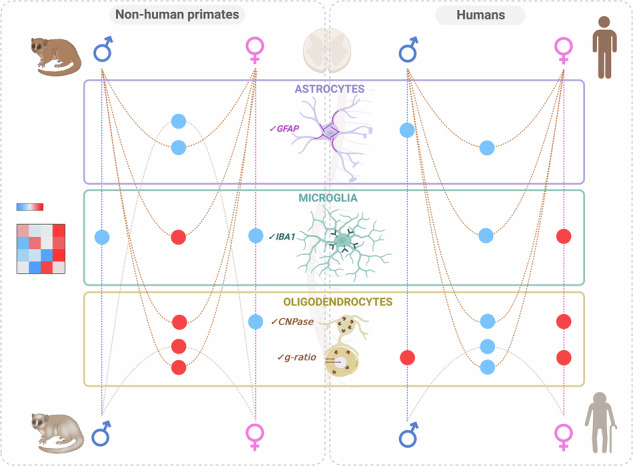

## Introduction

Reactive gliosis is a common response to central nervous system (CNS) pathologies, with glial responses primarily serving an evolutionarily conserved defensive role against pathological attacks and damages [for extensive review see [1]]. However, glial cells can also mediate harmful effects, including the excessive activation of a persistent inflammatory response. In the healthy CNS, genetic and epigenetic factors, including age, sex and spatial localization (different brain areas and the spinal cord [2]), influence glial phenotypes and functions, as well as their protective and detrimental responses to attacks.

Physiological glial aging is associated with morphological, cellular, molecular, and functional changes, generally reducing their neuroprotective roles. With age, astrocytes shrink in size, become less ramified and complex, lose their homeostatic and neuroprotective functions, and gain detrimental ones [for extensive review see, [1, 3]]. They also change their phenotype to feature shorter, thicker processes [for review see [4]], downregulate synaptic regulators, and upregulate cytokine signaling [5]. Over aging, microglia undergo changes in morphology, accumulate non-degraded inclusions, exhibit an enhanced inflammatory response, exhibit a compromised immune response, and show impaired motility [for detailed review see [6, 7]]. With age, white matter undergoes significant deterioration due to a decrease in oligodendrocyte numbers and a reduced capacity of oligodendrocyte precursor cells to proliferate and remyelinate [for review see [3]]. While myelin production continues with aging, it results in thinner myelin sheaths and shorter internodes [4].

Recently, sex-related factors in primates have gained attention in the study of differential physiological and pathological responses of glial cells. Transcriptomic data from purified human cortical astrocytes have identified sexual dimorphism at the molecular level, including genes encoding transcription factors and epigenetic regulators located on sex chromosomes [5]. These findings may have implications for sex differences in various neurological disorders. Additionally, the effects of sex on myelination indices have also been observed [8]. Single-cell analysis of human brain microglia revealed that microglia are organized into several types; however, these subtypes do not display sex differences [9]. Interestingly, spatial transcriptomics of the human spinal cord in midlife individuals (aged 35–59 years) highlighted sex differences in gene expression. The most significant differences in astrocytes, microglia, and oligodendrocytes were linked to sex-specific genes involved in X chromosome inactivation [10].

Few studies have examined age-dependent and sex-related changes in glia and myelin, particularly in primates, including humans. Due to their close physiological and genetic similarities to humans and their long lifespans, nonhuman primates (NHP) are invaluable for understanding aging-related changes that likely mirror those in humans. Notably, most research on glia and myelin has focused on the brain rather than the spinal cord. Among NHP, the grey mouse lemur (*Microcebus murinus*) has significantly contributed to our understanding of aging mechanisms [for review, see [11]]. Our research indicates that the adult spinal cord of *Microcebus murinus* shares more similarities in spinal cord architecture, glial components and myelin with humans than with mice [12, 13].

In this study, we investigated sex- and age-dependent differences in the healthy spinal cords of *Microcebus murinus* and humans. We analyzed specific glial markers and myelin characteristics, such as the g-ratio, in midlife and older individuals of both sexes. Our findings reveal that primate spinal cords exhibit distinct variations in glial markers expression and myelin characteristics based on sex and age, although these differences vary between species.

## Results

### The spinal cord of nonhuman primates exhibits sex and age differences in glial marker

**Fig. 1:**
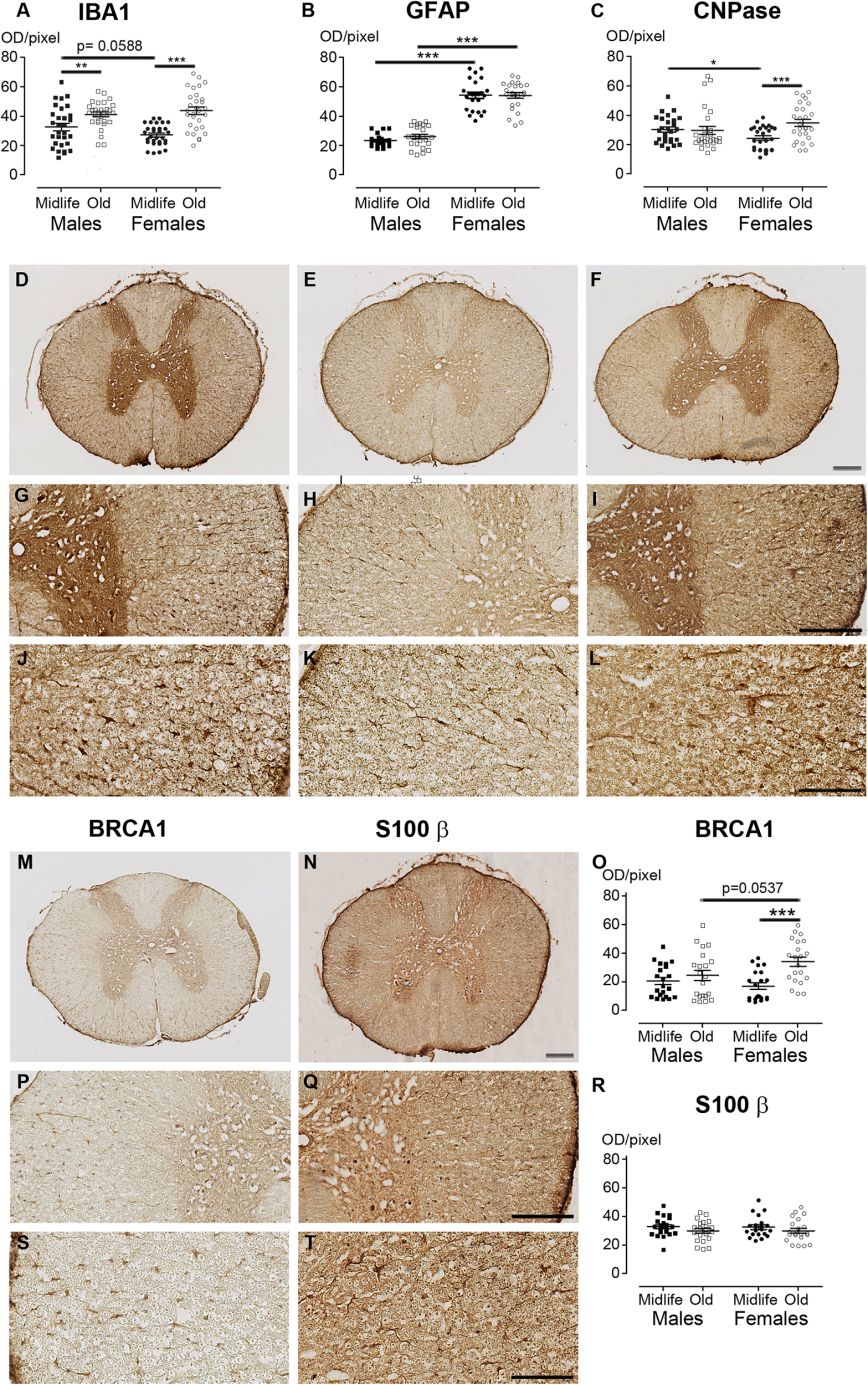
glial markers in the nonhuman spinal cord display sex and age dimorphisms. Quantification of the expression of IBA1 (**A**), GFAP (**B**), CNPase (**C**), BRCA1 (**O**) and S100β (**R**) in midlife and old males and females. Representative brightfield micrographs of IBA1 (**D,** **G** & **J**), GFAP (**E,** **H** & **K**), CNpase (**F,** **I** & **L**), BRCA1 (**M,** **P** & **S**) and S100β (**N,** **Q** & **T**) expression in the nonhuman primate spinal cord. Sections from the same animal, an old male, are shown for all staining (**D**-**L,** **N,** **Q** & **T)**, except for BRCA1, where sections from an old female are presented in (**M,** **P** & **S**). Three different magnifications are shown for each marker. Scale bars (**D-I,** **M-N** and **P-Q**): 200 µm; (**J-L** and **S-T**): 100 µm. Statistics *p < 0.05, **p < 0.01 and ***p < 0.001, un-paired t-test. Lemurs: *n* = 3 animals per group. For each individual, we quantified 10 spinal cord sections for IBA1, 8 sections for GFAP, 9 sections for CNPase, 7 sections for BRCA1 and S 100β. Sections were 14 µm thick and spaced 630 µm apart.

**Fig. 2:**
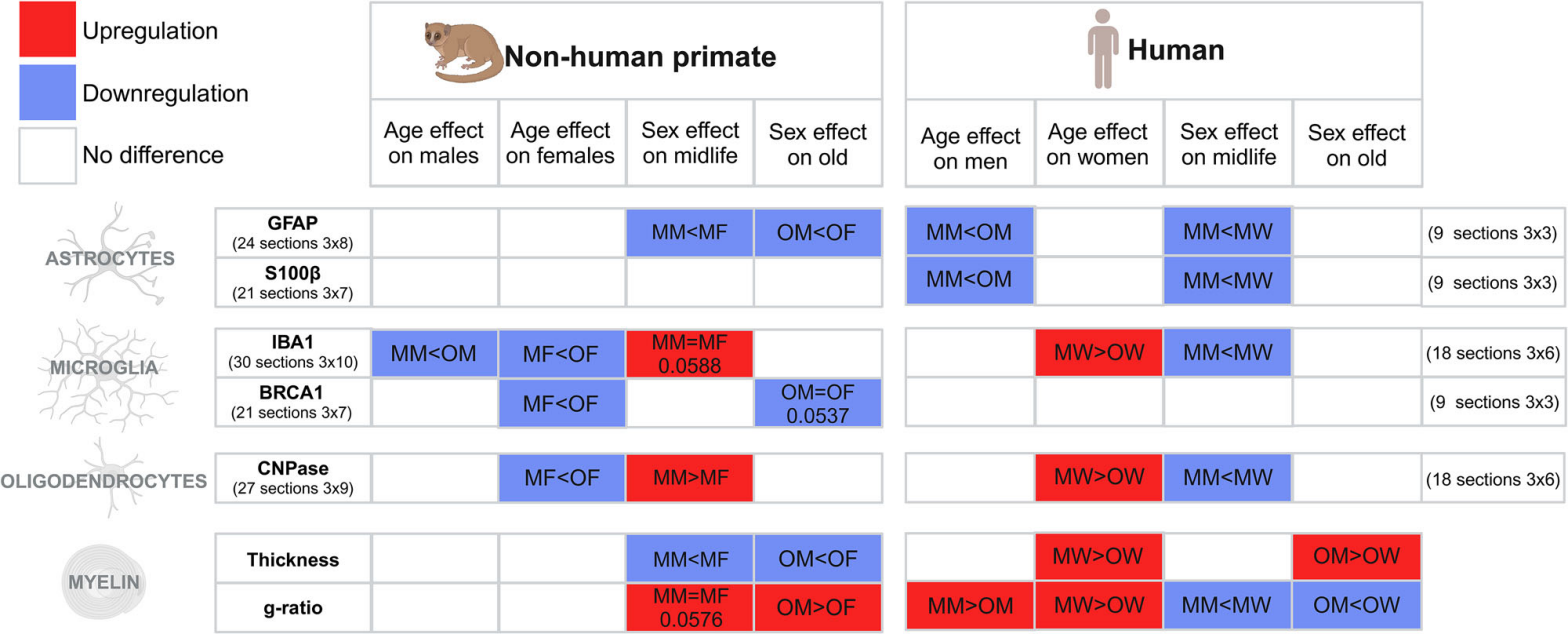
Levels of expression of glial markers and myelin characteristics in nonhuman primate and human. Comparison of the expression levels of astrocytic markers (GFAP & S100β), microglial markers (IBA1 & BRCA1), the oligodendrocytic marker (CNPase), and myelin characteristics (thickness & g-ratio) in nonhuman primates and humans of both sexes at midlife and old age. The abbreviations used are: MM midlife males/men, MF midlife females, MW midlife women, OM old males/men, OF old females, and OW old women.

In the spinal cord, we have previously shown that microglia and astrocytes display similar cell distribution and morphology in *Microcebus murinus* and human [12]. The lifespan of this species ranges from 8 to 12 years. Morphological changes indicative of aging, such as bleaching of the fur on the face, belly, and back, shortening of the snout, and thickening of the ear auricle border, typically appear at 5–6 years of age [11]. Thus, mouse lemurs younger than 5 years are considered adults, while those older than 6 years are regarded as aged animals. To investigate whether the expression of glial markers is sex-dependent and modulated over lifetime in nonhuman primates (NHP), we used selective microglial (IBA-1 and BRCA1), astrocytic (GFAP and S100β) and oligodendrocytic (CNPase) immunostainings. We analyzed six animals per sex [three midlife and three old animals]. The mean ages for midlife individuals were of 4.90 + /−0.08 and 5.14 + /−0.37 years for females and males, respectively. Old animals were aged 9.26 + /−0.46 and 9.44 + /−0.59 years for females and males, respectively. We examined the white matter (excluding the dorsal *funiculus*) of low thoracic (T10−T12) spinal cords. In both females and males, IBA-1 expression (Fig. 1A, D, G & J) showed age-dependent patterns, with midlife animals (Figs. 1A and 2) displaying lower expression compared to aged ones. We have previously shown that BRCA1 is expressed by microglia [14]. Similar to IBA-1, BRCA1 expression (Fig. 1M, P & S) was up-regulated by age in females (Figs. 1O and 2**)**. GFAP-expression (Fig. 1B, E, H & K) was sex-dependent. For both midlife and old individuals, the expression level of the astrocytic marker was lower in males than in females (Figs. 1B and 2). We observed no difference between groups using S100β as an alternative for astrocytic characterization (Figs. 1N, Q, R & T, and 2). Finally, we highlighted an age-dependent increase in CNPase expression (Fig. 1F, I & L) only in females (Figs. 1C and 2).

Furthermore, midlife animals exhibited a sex-dependent CNPase expression, with higher levels in midlife males (Figs. 1C and 2). Although using the mean value per individual reduces the number of significant results (Supplementary Fig. 1A–E), GFAP expression remains sex-dependent, with higher levels observed in females.

Together, these results show that NHP present sex-and age-dependent expression of glial markers. The variation between sexes and ages is more pronounced for microglia than for astrocytes and oligodendrocytes.

### The spinal cord of nonhuman primates exhibits sex and age differences in myelin

**Fig. 3:**
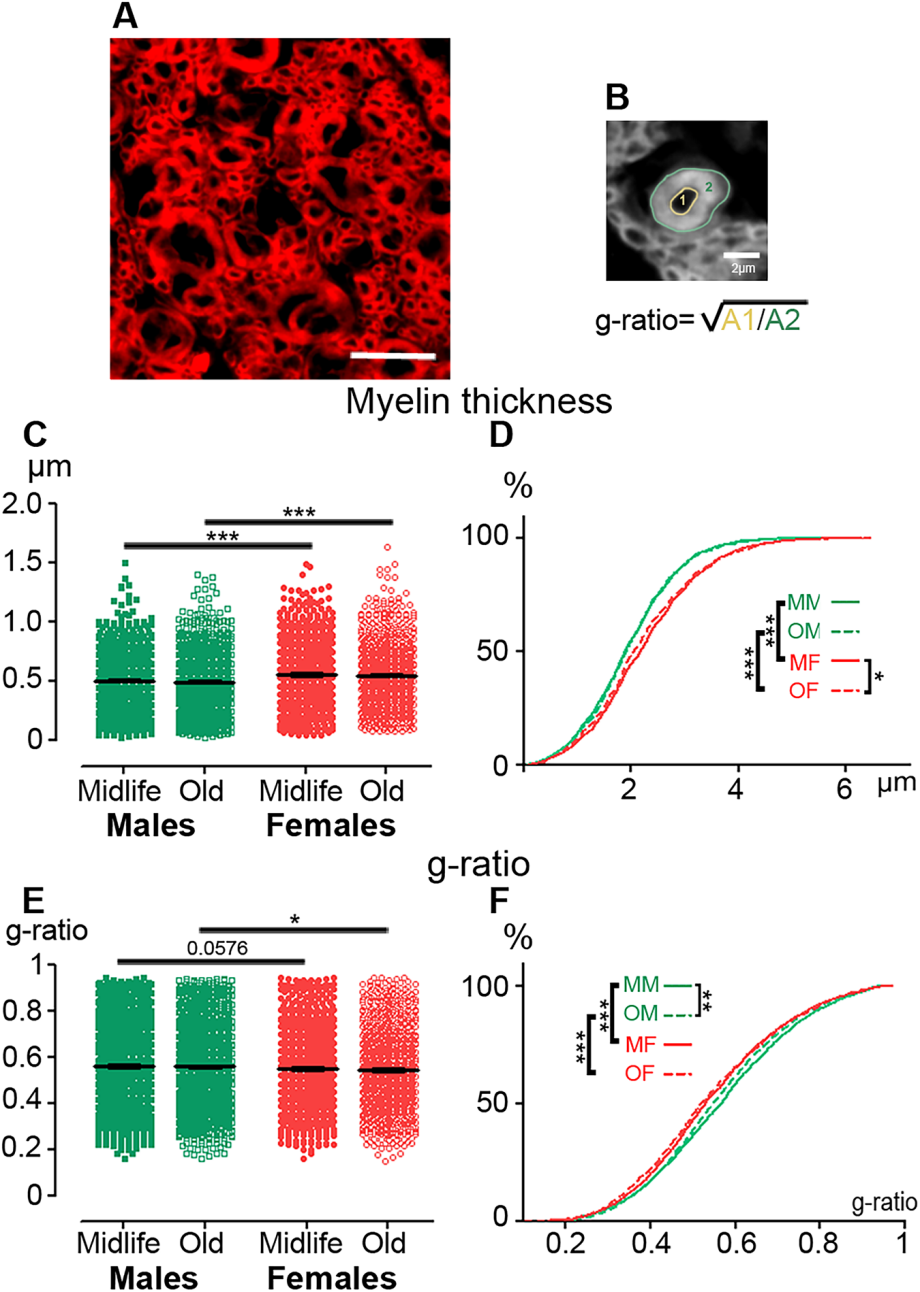
Myelin microstructure in the nonhuman spinal cord displays sex and age dimorphisms. Photograph of fluoromyelin staining of an axial section of a NHP thoracic spinal cord (**A**). Quantification method used to measure axon and fiber [2] areas and calculate the g-ratio (**B**). Quantification of myelin thickness (**C**) and g-ratio (**E**). Sample distributions of myelin thickness (**D**) and g-ratio (**F**). Scale bar 10 µm. Statistics *p < 0.05, **p < 0.01 and ***p < 0.001, un-paired *t*-test (**C** & **E**), Kolmogorov-Smirnov test (**D** & **F**). Lemurs: *n* = 3 in each group, for each individual we quantified at least 3 spinal cord sections of 14 µm separated from 630 µm and 200 fibers per individual.

To further investigate the microstructure of the spinal cord, with a particular focus on myelin, we quantified myelin thickness and g-ratio using spinal cord sections stained with fluoromyelin (Fig. 3A). The g-ratio is a geometric measure of axons that quantifies the degree of myelination in relation to their cross-sectional size (Fig. 3B) and is functionally relevant due to its crucial role in determining neuronal conduction velocity. Myelin thickness displays a sex dimorphism, with greater thickness in females compared to males in both midlife and old individuals (Figs. 3C and 2). The sample distribution, tested using the Kolmogorov–Smirnov statistic, showed age dependence in females and sex dependence in both midlife and old individuals (Figs. 3D and 2). The g-ratio similarly displays a sex dimorphism, with lower values in females compared to males in the elderly, and it nearly reaches significance (*p* = 0.0576) in midlife individuals (Figs. 3E and 2). The sample distribution, showed age dependence in males and sex dependence in both midlife and old individuals (Fig. 3F). These results show that NHPs exhibit sex-and age-dependent characteristics of myelin.

### The human spinal cord displays sex and age dimorphisms in the expression of glial markers

**Fig. 4:**
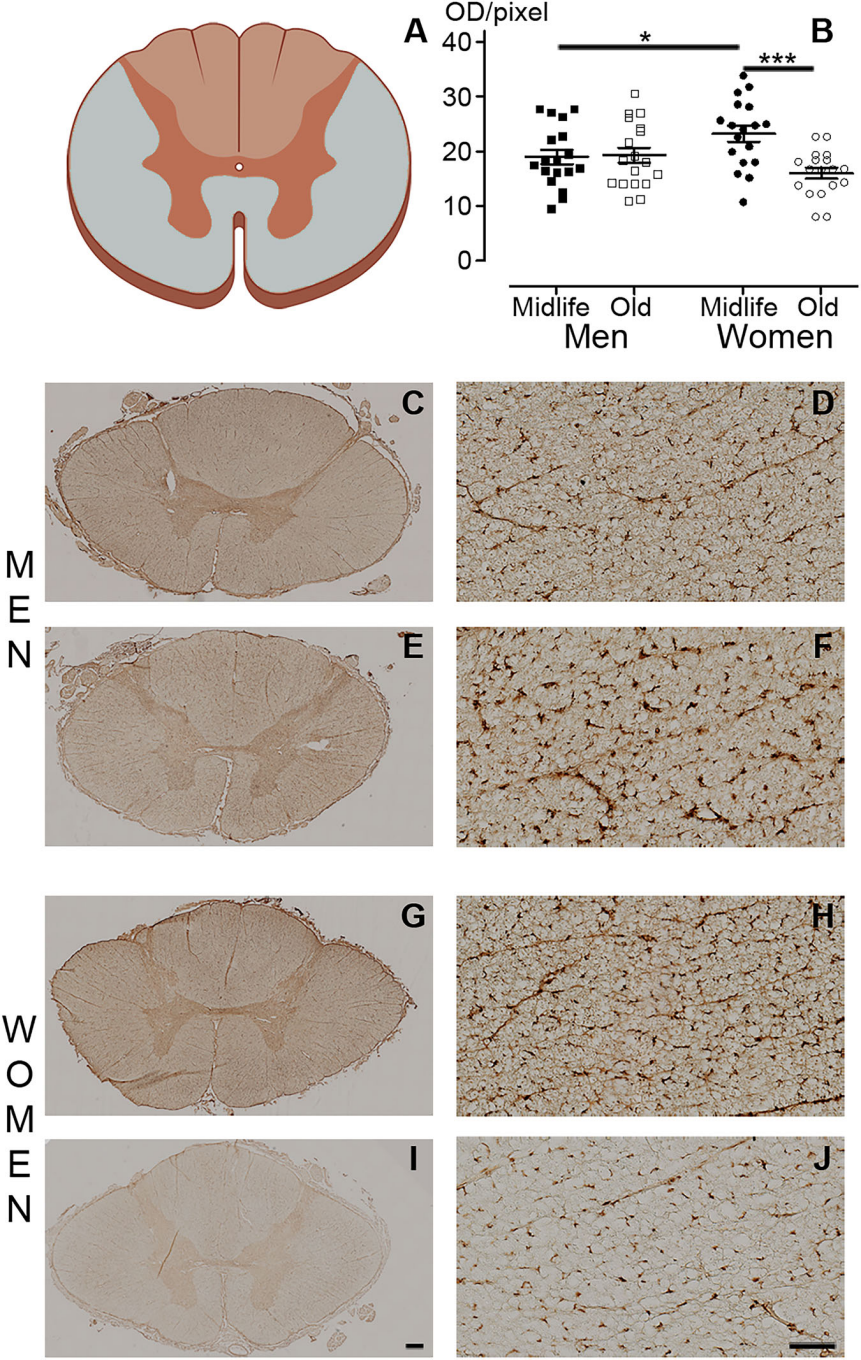
IBA1expression in the human spinal cord displays sex and age dimorphisms. Schematic view of the spinal cord and location of the quantification (white matter, excluding the dorsal *funiculus*) (**A**, grey area). Quantification of IBA1 expression (**B**). Representative brightfield micrographs of IBA1 expression in spinal cord at a low thoracic level in men (**C**-**F**) and women (**G**-**J**) in both midlife (**C**-**D** & **G**-**H**) and older (**E**-**F** & **I**-**J**) individuals. Higher magnifications (**D,** **F,** **H** & **J**) of (**C,** **E,** **G** & **I**), respectively. Scale bars (**C,** **E,** **G** & **I**): 500 µm and (**D,** **F,** **H** & **J**) 100 µm. Statistics *p < 0.05 and ***p < 0.001, un-paired *t*-test. Human: *n* = 3 individuals per group. For each individual, we quantified 6 spinal cord sections. Sections were 14 µm thick and spaced 154 µm apart.

**Fig. 5:**
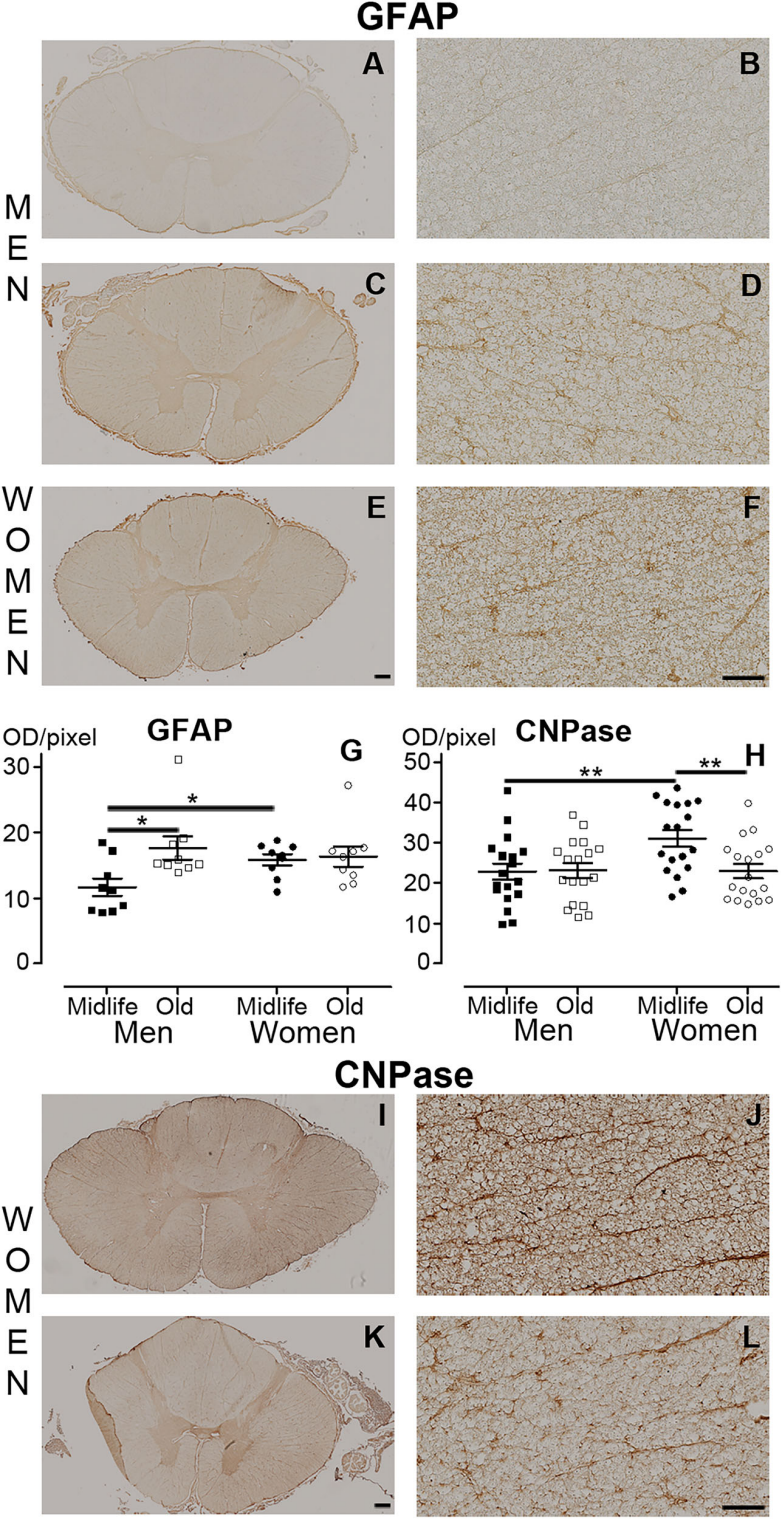
GFAP and CNPase expressions in the human spinal cord display sex and age dimorphisms. Representative brightfield micrographs of GFAP (**A**-**F)** and CNPase (**I**-**L**) expression in the human spinal cord at a low thoracic level. Higher magnifications (**B,** **D,** **F,** **J** & **L**) of (**A,** **C,** **E,** **I** & **K**), respectively. Midlife (**A**-**B**) and aged (**C**-**D**) men. Midlife (**E**-**F,** **I-J**) and aged (**K**-**L**) women. Quantification of GFAP (**G**) and CNPase (**H**) expressions. Scale bars (**A,** **C,** **E,** **I** & **K**): 500 µm and (**B,** **D,** **F,** **J** & **L**) 100 µm. Statistics *p < 0.05 and **p < 0.01, un-paired *t*-test. Human: *n* = 3 individuals per group. For each individual, we quantified 3 spinal cord sections for GFAP, and 6 for CNPase. Sections were 14 µm thick and spaced 154 µm apart.

We then conducted a comparative analysis using human spinal cords obtained from brain-dead organ donors. Specifically, we examined the white matter (excluding the dorsal *funiculus*) of low thoracic (T10-T12) spinal cords (Fig. 4A) from 6 women and 6 men, with 3 individuals of each sex in both midlife and old age groups. The mean age of midlife individuals was 53.67 + /−2.62 for women and 57.33 + /−4.51 years for men. In contrast, elderly individuals were aged 78.67 + /−3.09 years for women and 77.0 + /−5.57 years for men. In women, IBA-1 expression in the white matter (Fig. 3G-J) showed age dependence, with midlife individuals (Figs. 4B, G & H and 2) exhibiting higher expression than the elderly (Figs. 4B, I & J and 2). No age difference was observed for men (Figs. 4B-F and 2). Additionally, midlife individuals displayed sex-dependent IBA-1 expression with lower expression in men than in women (Figs. 4B, C-D & G-H and 2). No difference was observed between groups with BRCA1 expression (Fig. 2 and Supplementary Fig. 2A-C). GFAP-expression was age-dependent in men only (Figs. 5A-D & G and 2), with midlife men displaying lower expression than aged individuals (Figs. 5G and 2). No age difference was observed among woman groups (Figs. 5E-F & G and 2). Furthermore, midlife men exhibited lower expression of GFAP than midlife women (Figs. 5A-B, E-F & G, and 2). Similar results were confirmed using S100β, another astrocytic marker (Fig. 2 and Supplementary Fig. 2D-H). CNPase expression was age-dependent in women, with midlife individuals (Figs. 5H-J and 2) showing a higher expression levels of the oligodendrocytic marker than the elderly (Figs. 5H, K-L, and 2). Additionally, we observed a sex-dependent level of CNPase expression in midlife individuals, with lower expression in men than in women (Figs. 5H and 2). Although using the mean value per individual reduces the number of significant results (Supplementary Fig. 2f–J), IBA1 expression remains age-dependent in women, with higher levels observed in midlife individuals and S100β expression remains sex-dependent in midlife individual, with lower levels observed in men.

In summary, these results demonstrate that the human spinal cord exhibits sex-and age-dependent expression of glial markers.

### The human spinal cord displays sex and age dimorphisms in myelin

**Fig. 6:**
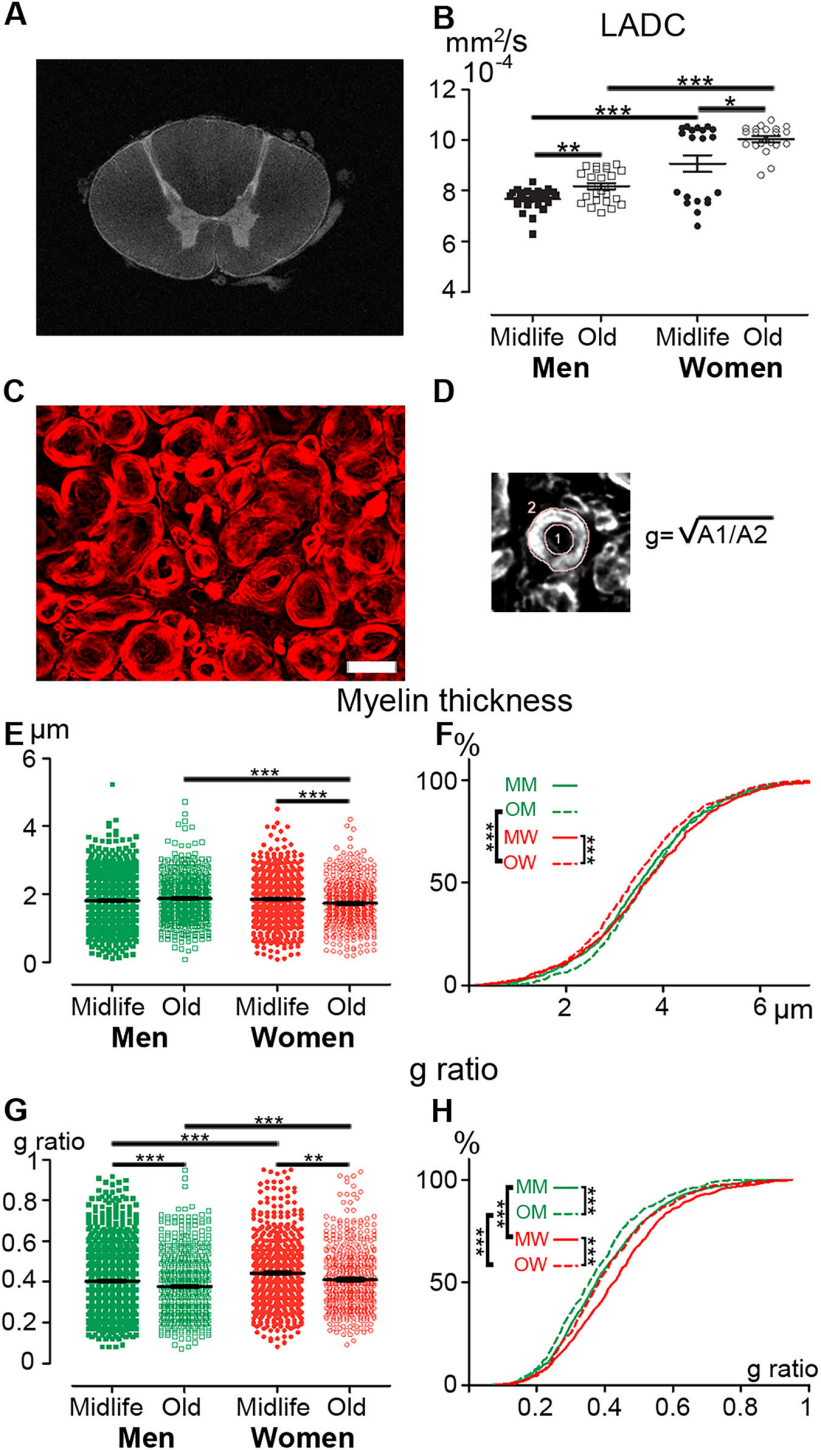
Myelin microstructure in the human spinal cord displays sex and age dimorphisms. Ex vivo diffusion-weighted MRI of the human spinal cord of a midlife woman at thoracic level (**A**). Quantification of the ex vivo DW-MRI longitudinal apparent diffusion coefficient (LADC) in the white matter in midlife and aged humans of both sexes (**B**). Photographs of fluoromyelin staining of an axial section of a human thoracic spinal cord (**C**). Quantification method used to measure axon and fiber areas and calculate the g-ratio [g] (**D**). Quantification of myelin thickness (**E**) and g-ratio (**G)**. Sample distributions of myelin thickness (**F**) and g-ratio (**H)**. Scale bar 10 µm (**C**). Statistics *p < 0.05, ** *p* < 0.01 and ***p < 0.001, un-paired *t*-test (**E**–**G**), Kolmogorov-Smirnov test (**F**–**H**). Human: *n* = 3 in each group, for each individual we analyzed 3 spinal cord sections of 14 µm separated from 154 µm and at least 50 fibers.

To further investigate the microstructure of the myelin spinal cord, we firstly employed ex vivo diffusion-weighted magnetic resonance imaging (DW-MRI), allowing for the clear identification of white and grey matter (Fig. 6A). We quantified the longitudinal apparent diffusion coefficient (LADC) in the white matter along a 2 cm-spinal cord segment (Fig. 6B). The LADC demonstrated age and sex dependencies; in both sexes midlife individuals displayed lower LADC than the elderly (Fig. 6B). Moreover, within each age group, men exhibited lower LADC than women (Fig. 6B). Secondly, we quantified myelin thickness and g-ratio (Fig. 6D) using spinal cord transverse sections stained with fluoromyelin (Fig. 6C). In women, myelin thickness showed age dependence, with midlife individuals exhibiting thicker myelin sheets than the elderly (Figs. 5E, and 2). No age difference was observed for men (Figs. 6E and 2). Additionally, aged individuals displayed sex-dependent myelin thickness with finer myelin sheets in women than in men (Figs. 6E and 2). Similarly, samples distribution, tested by Kolmogorov–Smirnov statistic, showed age dependence in women and sex dependence in the elderly (Fig. 6F).

Finally, the g-ratio exhibited age and sex dependencies. In both sexes, older individuals displayed a lower g-ratio than midlife individuals (Figs. 6G and 2). At both ages, women showed a higher g-ratio than men (Figs. 6G and 2). Likewise, samples distribution showed age and sex dependencies (Fig. 6H).

In summary, these results indicate that the human spinal cord displays sex-and age-dependent variations in myelin microstructure.

### Glial and myelin characteristics display sex and age dimorphisms in both species

In both species, some astrocytic markers display similar patterns across sex and age (Fig. 2). GFAP expression is lower in midlife males compared to older males in humans only. However, in both species midlife males express less GFAP than midlife females. Moreover, in NHP in older individuals, males also express less GFAP than females. In humans, conversely to NHP, similar results are obtained using GFAP and S100β. Deregulation of the microglial marker IBA1 is not consistent across sex and age in both species (Fig. 2). In NHP, IBA1 expression is lower in midlife individuals and higher in midlife males compared to midlife females. BRCA1, the other microglia marker displays an age-dependent expression in females. In humans, IBA1 expression is higher in midlife women compared to older individuals, and midlife men display lower expression than midlife women. No difference is observed for BRCA1 expression. Similarly, deregulation of the oligodendrocytic marker CNPase and myelin characteristics are not consistent across sex and age in both species (Fig. 2). In NHP, CNPase expression is lower in midlife females compared to old females and midlife males display higher expression than midlife females. The opposite is observed in humans, with higher expression in midlife women compared to elderly women, and midlife men displaying lower expression than midlife women (Fig. 2). In NHP, males display a lower myelin thickness and a higher g-ratio than females across ages (Fig. 2). In humans, myelin thickness is higher in midlife women compared to old women, and old men display thicker myelin than old women (Fig. 2). In humans, the g-ratio is higher in midlife individuals compared to older individuals, and smaller in men compared to women across ages (Fig. 2).

In summary, these results indicate that the primate spinal cord exhibits sex- and age-dependent variations in glial markers and myelin microstructure.

**Fig. 7:**
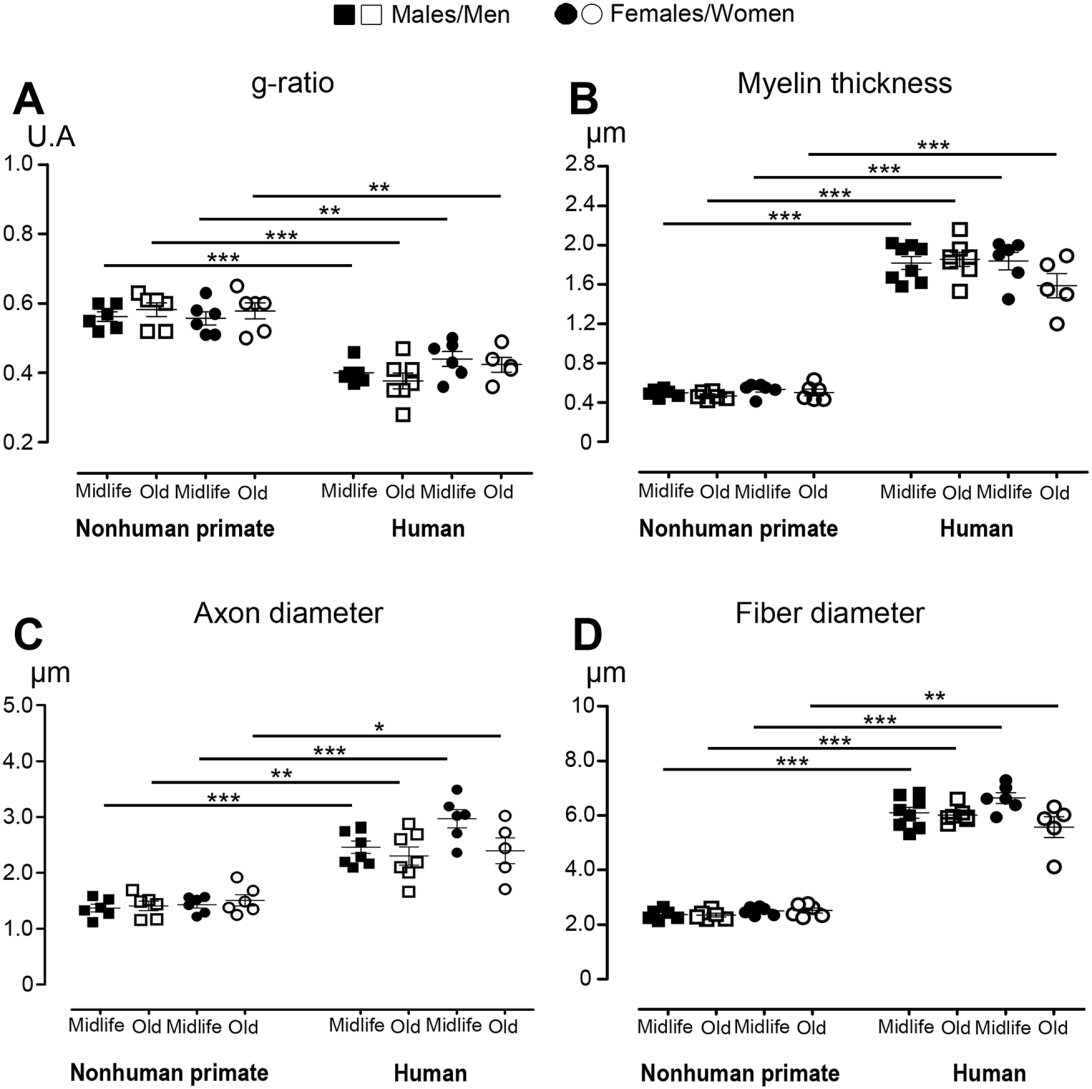
Comparison of myelin characteristics between nonhuman primates and humans. All comparisons between NHPs and humans were conducted on identical groups (age and sex) only. Calculation of g-ratio values (**A**). Quantification of myelin thickness (**B**). Measurement of axon diameter (**C**). Measurement of fiber diameter (**D)**. Statistics: mean values of each lateral *funiculus* per individuals were used, *p < 0.05, **p < 0.01 and ***p < 0.001, un-paired *t*-test (**A**–**D**).

As the analysis of fluoromyelin does not require that the experiments be conducted simultaneously in both species, we carried out a direct comparison of myelin characteristics (Fig. 7). A comparison of mean values from each lateral *funiculi* per individual highlighted that differences between species are consistent across ages in both sexes. The g-ratio is higher in NHPs than in humans (Fig. 7A), which is associated with lower myelin thickness (Fig. 7B), axon diameter (Fig. 7C), and fiber diameter (Fig. 7D) in NHPs compared to humans.

## Discussion

Our results show that NHP and human spinal cords display notable differences in glial markers expression and myelin characteristics depending on sex and age, with these variations differing between species.

### The spinal cord of human shows an increase in astrocytic markers with age in males

In *Microcebus murinus*, males express less GFAP than females across age. Interestingly, a previous study in aged *Microcebus murinus* (>7 years old, both sexes), found that increased GFAP expression was associated with brain atrophy [15]. Marmosets and vervets also show increased GFAP expression with age (for review see [16]. A study spanning the entire lifespan of rhesus macaques (8 males and 11 females; 0.45–25.59 years old) demonstrated a decrease in astrocyte numbers in the white matter with aging, along with modified astrocytic morphology characterized by increased process length and complexity, which is indicative of activation [17].

In the human spinal cord, we found that GFAP and S100β expression increase with age in men. This finding is consistent with an earlier study involving 33 human brains ranging from ages 12 to 98, which also reported increased GFAP expression with age. This increase becomes particularly pronounced after age 65, specifically in the hippocampus and entorhinal cortex [18]. Assessment of neocortical glial cells in the human brain (using hematoxylin and eosin staining) across different ages in two groups [18 women (average age 65 years, range 18–93) and 13 men (average age 57 years, range 19–87)] showed that the number of astrocytes remained constant throughout life in both sexes [19]. In a recent study, human neocortical slices from young (22–50 years) and older (51–72 years) adults of both sexes showed an age-dependent decrease in astrocytic size and complexity associated with an upregulation of GFAP expression [20]. In addition to age-related morphological changes in astrocytes, such as atrophy, functional asthenia is also observed, reflecting the loss of critical homeostatic functions, including glutamate clearance. Furthermore, astrocytic atrophy may impair synaptic transmission. Notably, this combination of atrophy and functional decline is associated with the development of certain pathological neurological processes [20]. Increased GFAP expression was not accompanied by an increase in astrocyte numbers, suggesting that the elevated GFAP levels occurred within individual astrocytes. We find similar evidence in men, despite using different methodologies and examining a different CNS location. However, we observe no age-related modification of GFAP expression in women’s spinal cords, confirming that astrocytes exhibit region-dependent differences, as previously demonstrated in the brain [4, 5, 21]. Of note, as previously reported, GFAP is not a fully reliable marker of the complete morphology of astrocytes, as it does not label peripheral processes that account for a significant portion of their surface [1]. Moreover, GFAP expression can increase while astrocytic arborization and perisynaptic leaflets decrease, meaning that GFAP upregulation does not necessarily indicate astrocytic hypertrophy or functional improvement [1].

### The spinal cord of primates exhibits differences in microglial status based on species, sex and age

In *Microcebus murinus*, we found that IBA1 expression is lower in midlife individuals compared to older individuals, and that midlife males have higher IBA1 expression than midlife females. In a comprehensive review on aging primates, Freire-Cobo et al. noted that NHP exhibit glial modifications in the aging brain, with species-specific differences [16]. Chimpanzees and marmosets did not show age-related changes in glial densities, though morphological changes indicative of microglial activation were observed. Conversely, in *rhesus* macaques there was an increase in gray matter microglial density and a decrease in white matter microglial process length with age but no sex differences were observed [17].

In humans, we found that IBA1 expression decreases with age in women while midlife men exhibit lower IBA1 expression than women. This finding aligns with an assessment of neocortical glial cells in human, which revealed a decrease in microglia numbers with age, but only in women [19]. The same research team later reported a non-significant trend towards decreased microglia in women across three age groups (65–75, 76–85, and 86–105 years old) [22].

Our results in the primate spinal cord confirm that microglia show species-dependent modifications with age, consistent with previous findings in the brain [for review see [6]].

### In primates, oligodendrocyte markers and myelin in the spinal cord vary based on age and sex

In *Microcebus murinus*, we found that CNPase expression is lower in midlife females compared to elderly individuals. Additionally, midlife males had higher CNPase expression than midlife females. In contrast, in humans, we observed the opposite pattern: midlife women had higher CNPase expression than elderly women, and midlife men had lower CNPase expression than midlife women. These findings are in agreement with an assessment of neocortical glial cells in humans, which reported a 27% decrease in oligodendrocytes with age, with a stronger correlation to age in women than in men [19]. The same research team later reported a 34% decrease in oligodendrocytes in women across three age groups [22].

In *Microcebus murinus*, we found that myelin thickness and the g-ratio are sex-dependent across age but are not age-dependent within each sex. This contrasts with the reduction in myelin thickness and density observed in the *corpus callosum* of older marmosets compared to younger ones [16]. However, it is important to note that these studies involve two different NHP and two distinct areas of the CNS.

In human, we found an age-related decrease in myelin thickness in women and a sex-dependent difference in older individuals. We also observed that the g-ratio strongly correlates with both age and sex. This contrasts with MRI-based estimates of the g-ratio in the human brain, which show that the g-ratio is correlated with age but not with sex [23, 24]. This discrepancy in sex-dependency is likely influenced by the methods used to calculate the g-ratio (fluoromyelin staining *versus* MRI), as well as the specific CNS region studied (spinal cord *versus* brain). The most likely interpretation is that the subtle reduction in myelin thickness in the spinal cord over the lifespan is less pronounced in men than in women. This difference may contribute to the sexual dimorphism observed in multiple sclerosis [25].

A direct comparison of the g-ratio of myelin fibers in the lateral *funiculus* confirmed and extended our previous findings to both sexes and across ages, showing a higher g-ratio in NHPs than in humans, which inversely correlates with species evolution [13]. This difference may partly reflect variations in fiber conduction speed across species.

### Glial cells of the primate spinal cord exhibit age and sex differences: implications for pathologies

It is well established that age and sex significantly affect the prevalence and underlying mechanisms of CNS diseases, including those involving the spinal cord. Although recent research has focused on the role of glial cells in these processes, the impact of sexual dimorphism on spinal cord diseases has not been extensively studied. This gap in research is largely due to the limited availability of primate spinal cord tissues, particularly human samples. However, a recent study highlighted sex differences in the gene expression of human spinal glial cells, especially in genes associated with multiple sclerosis, neuropathic pain, and amyotrophic lateral sclerosis [10].

In both species, we observe differences in glial marker expression and myelin characteristics based on sex, both in midlife and older individuals. Sex hormones and their metabolites influence astrocyte shape and GFAP expression [for review see, [26]], while estrogen affects the microglial inflammatory response [27]. Evidences indicate that there are sex-based differences in microglial morphology and phagocytic ability in the adult CNS [28], as well as in gene expression patterns [29]. Additionally, sex differences in white matter have been reported in humans [30, 31]. Given these differences in the uninjured spinal cord, glial cells are certainly critical for age and sex differences in the prevalence and progression of spinal cord diseases, including neuropathic pain, multiple sclerosis and amyotrophic lateral sclerosis as well as their response to traumatic spinal cord injury [32].

## Conclusion

It is not entirely clear to what extent aging and sex affect the ability of glial cells to respond to spinal cord pathology or their role in the incidence and pathophysiology of spinal cord diseases. Further research is needed to elucidate age and sex-related characteristics of primate glial cells and myelin in the healthy spinal cord, and to understand the mechanisms regulating sex differences in glial responses across the lifespan. Recognizing that glial characteristics vary by age and sex is crucial, as these factors may influence differences in vulnerability, incidence, pathogenesis, and prognosis of neurological diseases, as well as affect the efficacy of potential therapies.

## Methods

### Spinal cord samples

Nonhuman primates: lemurs (*Microcebus murinus*) were born and bred in the animal facility (CECEMA, University of Montpellier, France; license approval 34-05-026-FS), housed in enriched cages in temperature and hygrometry-controlled environment (24-26°C, 55% of humidity). Animals were fed with fresh fruits and a mixture of cereal, milk and eggs; water was given ad libitum. For tissues collection, animals were injected with a lethal dose of ketamine (150 mg/kg, Merial, Lyon, France). Vertebral blocks were collected and immersed for 48 h in 4% paraformaldehyde (PFA, pH7.2, Sigma Aldrich, Darmstadt, Germany) in 0.1 M phosphate-buffered saline (PBS). Spinal cords were dissected and post-fixed 2 h in 4% PFA, incubated in 30% sucrose in 0.1 M PBS, frozen in OCT (Sakura, Alphen aan den Rijn, Netherlands) and stored at −20 °C. In captivity, the median survival time for *Microcebus murinus* is 4.9 years for females and 5.7 years for males [11]. Twelve lemurs were included in the study, 6 females [3 midlife animals, mean age 4.90 + /−0.08 years; 3 old animals, mean age 9.26 + /−0.46 years] and 6 males [3 midlife lemurs, mean age 5.14 + /−0.37 years; 3 old lemurs, mean age 9.44 + /−0.59 years]. We selected these age ranges to match those of the human spinal cord samples, with midlife individuals representing the onset of aging and aged individuals approximating life expectancy. Low thoracic (T10–T12) spinal cords were analyzed.

Humans: low thoracic (T10–T12) spinal cords were obtained from 12 brain-dead organ-donor under the approval of the French institution for organ transplantation. Six women [3 midlife women, mean age 53.67 + /−2.62 years; 3 older women, mean age 78.67 + /−3.09 years] and 6 men [3 midlife men, mean age 57.33 + /−4.51 years; 3 older men, mean age 77.0 + /−5.57 years]. One patient died from head gunshot, 4 from aneurysm and 7 from stroke. Sample collection was done as previously described [13, 33–35]. Briefly, body temperature was lowered and ventilation and blood circulation were maintained until approximately 4 h before spinal cord dissection. Immediately after organs removal for therapeutic purposes, T9–L4 vertebral blocks were extracted and dissected spinal cords were immediately fixed in 4% PFA. The short-time interval between ventilatory and circulatory arrest and sample fixation permitted a high tissue quality.

### Ex vivo diffusion-weighted magnetic resonance imaging (DW-MRI)

Human spinal cords were post-fixed in 4% PFA for at least 48 h and further stored in 1% PFA until ex vivo MRI acquisition. Spinal cords were placed in Fluorinert FC-40 liquid (3 M™ Electronic Liquids, Saint Paul, Minesota, USA) in a glass tube surrounded by a custom-made solenoid coil dedicated to SCI investigations [36, 37]. The coil was positioned in a 9.4 Tesla apparatus (Agilent Varian 9.4/160/ASR, Santa Clara, California, USA) associated with a VnmrJ Imaging acquisition system (Agilent, USA). Axial MRI scans were done using Single Echo Multi Slices (SEMS) sequence (TR = 1580 ms; TE = 30.55 ms; AVG = 30; FOV = 10 mm*10 mm; 36 slices; thickness = 1 mm; gap = 0 mm; acquisition matrix (NREAD*NPHASE) = 128*128). Diffusion gradients were applied in 3 directions including the rostro-caudal axis and 2 directions perpendicular to the spinal cord (Gs = 10 G/cm; delta = 6.844 ms; separation = 15.05 ms; b-value = 499.21 s/mm²). Images were also acquired without diffusion gradient (Gs = 0 G/m^−1^) as described previously [38]. Segmentations were done manually and longitudinal and transversal apparent diffusion coefficients were measured (ImageJ, National Institutes of Health, USA). Just after MRI scans, samples were rinsed in PBS, cryoprotected in 30% sucrose, embedded in Tissue Tek (Sakura, Alphen aan den Rijn, The Netherlands), frozen and kept at −20°C.

### Histology and immunohistochemistry

Serial 14-micrometer (µm)-thick axial spinal cord cryosections (Microm HM550, Thermofisher Scientific, Waltham, Massachusetts, USA) were collected on Superfrost Plus©slides. Sections were analyzed at 630 µm intervals in NHP and 154 µm intervals in human.

#### Histology

To visualize myelin fibers, we used fluoromyelin staining. As previously described [13, 38, 39], cryosections from both species were incubated 20 min with fluoromyelin (1:200, Invitrogen, Carlsbad, California, USA), rinsed 3×10 min in PBS and mounted with fluorosave (Dako, Glostrup, Denmark).

#### Immunohistochemistry

14-µm-thick axial spinal cord sections were washed twice in 0.1 M PBS, dipped for 10 min in 0.1 M PBS containing 20 mM lysine (pH 7.2) followed by 30 min in hydrogen peroxide (1% in 0.1 M PBS, Sigma Aldrich, Gilligham, UK), washed twice in 0.1 M PBS, blocked for 2 h with 0.1 M PBS containing 1% bovine serum albumin and Triton X-100 (0.1%) (both from Sigma Aldrich, Gilligham, UK) and incubated with the primary antibody for 48 h at 4°C (in blocking buffer). Negative controls were done without primary antibody. Sections were rinsed for 30 min with 0.1 M PBS, incubated in the corresponding secondary antibody for 2 h at room temperature (RT) and rinsed with 0.1 M PBS (30 min). For amplification (IBA1, BRCA1 and CNPase in both species and GFAP in NHP), we used Avidin Biotin Complex solution (Vector Laboratories Ltd. Peterborough, UK) (1:100 in 0.1 M PBS, 1 h, RT) and sections were rinsed in 0.1 M Tris (pH 7.6, RT). We used DAB peroxidase substrate kit (Vector Labs, Burlingame, California, USA) to visualize protein expression and we rinsed the sections in 0.1 M Tris (3×10 min) to stop the reaction. Slides were dehydrated in increasing concentrations of ethanol and then cleared with xylene. Coverslips were applied using Eukitt (Sigma Aldrich, Darmstadt, Germany). All immunostainings for a given species and a given antibody were done simultaneously to minimize bias in staining intensity.

#### Antibodies

Primary antibodies: rabbit anti-glial fibrillary acidic protein (GFAP, 1:500, #Z0334; Dako, Denmark), rabbit anti-ionized calcium-binding adapter molecule 1 (IBA1, 1:200; #PTR2404, Wako Pure Chemical Industries, Japan), mouse anti-2’,3’-Cyclic-nucleotide 3’-phosphodiesterase (CNPase, 1:400, #C5922, Sigma Aldrich, Gilligham, UK) mouse anti-BReast CAncer gene 1 (BRCA1, 1:100, Santa Cruz Biotechnology, #SC6954, Inc., Dallas, Texas, USA) and rabbit anti- S100b (1:500, #Z0311, Dako, Glostrup, Denmark).

Secondary antibodies: corresponding biotinylated antibodies (1:500, Invitrogen, Carlsbad, #31821, California, USA) or anti-rabbit (1:500 Thermo Fisher Scientific, #31458, Waltham, MA USA).

#### Microscopy and quantifications

Brightfield slide scans were obtained using NanoZoomer scanner (NanoZoomer Digital Pathology System, Hamamatsu, Japan) with constant light intensity and exposure time.

To quantify protein expression (IBA1, BRCA1, GFAP, S100β and CNPase), the mean optical density (OD) was measured in transverse sections at different distance along the rostro caudal axis of a spinal cord thoracic segment (lemur or human), as in our previous studies [12, 37, 38, 40, 41]. OD is a robust and precise method for quantifying protein expression levels and identifying changes under different experimental conditions. OD quantifications were performed in the same regions for both species, specifically in the white matter (excluding the dorsal *funiculus*) (Fig. 4A) at the lower thoracic spinal cord level (T10–T12). Digital images (NDP view software, Hamamatsu, Japan) were exported using identical parameters and settings. For each section, the mean signal intensity of the adjacent non-specific background was determined by selecting an area devoid of tissue and then subtracted from the mean signal intensity of the stained region of interest. For each comparison performed for a given staining in a given species, we used the same number of sections per group. For all antibodies used, expression levels were analyzed in at least 7 axial sections at 630 µm intervals per NHP and 3 axial sections at 154 µm intervals per human. All quantifications were done blindly using ImageJ (National Institutes of Health, USA).

Fluorescent images (fluoromyelin) were acquired with THUNDER Imager 3D (Leica, Wetzlar, Germany; lens X 63). A 5 µm-thick/24 slices stack (z-step: 0.21 µm) of 600×400 µm field was acquired in both lateral *funiculi* at the lower thoracic spinal cord level (T10–T12) for both species. Stacks were cleared using the THUNDER imager 3D Large Volume Computational Clearing process and a single-slice field (mid-stack) was exported. 20 fields (40×40 µm) were exported in both lateral *funiculi* for quantification. Intact myelin fibers parameters (g-ratio, myelin thickness and fiber diameter) were determined using a home-developed macros with Fiji [42] as previously described [39]. Briefly, the analysis begins by manually selecting the center of each myelin fiber (corresponding to the axon) in the image (Supplementary Fig. 3). A local threshold is then applied using Phansalkar’s algorithm [43], creating a binary image where the myelin appears in white and the axons in black. The Wand tool is used to identify the black region corresponding to the manually selected point, representing the axon. To measure the surrounding myelin, the black region is progressively enlarged until it reaches the boundary of the surrounding white area, stopping at another black region (either an adjacent axon or background). The myelin area is then defined by subtracting the axonal area from the total region. To ensure measurement accuracy, we verified the myelin segmentation (Supplementary Fig. 3B-C), and if it was not accurate (Supplementary Fig. 3D), we removed the selected fiber. We determined myelin fiber parameters such as the g-ratio, myelin thickness and fiber diameter in at least 200 fibers per nonhuman primate and 50 fibers per human.

#### Artwork and statistics

Images were assembled using Photoshop (Adobe, San Jose, California, USA). Statistical tests were done using GraphPad Prism version 5.03 (GraphPad software, Boston, Massachusetts, USA) and significance was accepted at *p* ≤ 0.05. Results are expressed as mean ± standard error of the mean (SEM). All comparisons between groups (fluoromyelin, immunohistochemistry and MRI) were done using un-paired *t*-test with Welch correction. The Kolmogorov–Smirnov test was used to compare the distribution between groups of myelinated fibers based on their thickness or g-ratio (https://www.aatbio.com/tools/kolmogorov-smirnov-k-s-test-calculator).

Due to the rarity of primate (human and NHP) samples, we had access to only three individuals per group. A power analysis indicated that 21 samples are needed to achieve adequate statistical power. All immunostainings for a given species and antibody were performed simultaneously to minimize bias in staining intensity. The detachment or folding of some sections made it impossible to analyze the same number of sections across all groups. To minimize potential bias from varying numbers of measurements between groups for a given species and staining, we standardized the number of analyzed sections. Therefore, for each experimental condition, we analyzed at least seven sections.

## Supplementary information

Legends for Supplementary Figures

Supplementary Figure 1 : Mean expression levels of all markers in the nonhuman primate and human spinal cord

Supplementary Figure 2 : BRCA1 and S100β expression in the human spinal cord to further characterize glial cells

Supplementary Figure 3: Method for quantifying the myelin g-ratio

## Data Availability

All data analyzed and computer code designed during this study are included in the published article and its supporting information are available from the corresponding author on reasonable request.

## Code availability

All data analyzed and computer code designed during this study are included in the published article and its supporting information are available from the corresponding author on reasonable request.
