# Supplementary material for: Sex and age differences in glia and myelin in nonhuman primate and human spinal cords: implications for pathology
Source: Cell Death Discov. 2025 Apr 2;11:129. doi: 10.1038/s41420-025-02425-9 (PMC11965325; doi:10.1038/s41420-025-02425-9)
Supplement: Supplementary file 1 — Legends for Supplementary Figures [file 41420_2025_2425_MOESM1_ESM.docx]

**Sex and Age Differences in Glia and Myelin in Nonhuman Primate and Human Spinal Cords: Implications for Pathology**

Gaëtan Poulen, MD/PhD*, Nacéra Douich, MSc.*, Chloé M. Gazard, MSc., Nadine Mestre-Francés PhD, Maïda Cardoso, MSc., Luc Bauchet MD/PhD, Florence Vachiery-Lahaye MD, Nicolas Lonjon MD/PhD, Yannick N. Gerber PhD and Florence E. Perrin PhD^†^

**SUPPLEMENTARY FIGURE LEGENDS**

**Supplementary Figure 1: Mean expression levels of all markers in the nonhuman primate and human spinal cord**

Quantification of glial markers in NHP (**A**-**E**) and human (**F**-**J**) low thoracic spinal cord transverse sections. Mean expression levels of IBA1 (**A**&**F**), GFAP (**B**&**G**), CNPase (**C**&**H**), BRCA1 (**D**&**I**) and S100β (**E**&**J**). The white matter, excluding the dorsal *funiculus*, was quantified in both species. Lemurs and human: n = 3 individuals per group. Sections were 14 µm thick and spaced 630 µm apart for lemurs and sections were 14 µm thick and spaced 154 µm apart for human. Statistics *: p<0.05 and **: p<0.01 un-paired t-test.

**Supplementary Figure 2: BRCA1 and S100β expression in the human spinal cord to further characterize glial cells**

Representative brightfield micrographs of BRCA1 expression in the low thoracic human spinal cord of midlife man (**A**-**B**). Zoomed image (**B**) of (**A**). Quantification of BRCA1 expression (**C**). Representative brightfield micrographs of S100β expression in the low thoracic human spinal cord of midlife woman (**E**-**F**) and aged man (**G**-**H**). Zoomed images (**F**&**H**) of (**E**&**G**), respectively. Quantification of S100β expression (**D**). Scale bars (**A**, **E**&**G**): 500µm and (**B**, **F**&**H**) 100µm. Statistics **: p<0.01, un-paired t-test. Human: n = 3 individuals per group. For each individual, we quantified 3 spinal cord sections for BRCA1 and S100β. Sections were 14 µm thick and spaced 154 µm apart.

**Supplementary Figure 3: Method for quantifying the myelin g-ratio**

Representative example of fluoromyelin staining in NHP (**A**). Example of myelin fibers detection using a home developed macro in FIJI (**B**). Accurate myelin selection and g-ratio measurement axon [1] and fiber [2] (**C**). Inaccurate myelin selection (**D**).
