# Supplementary figures and images for: Sex and age differences in glia and myelin in nonhuman primate and human spinal cords: implications for pathology
Source: Cell Death Discov. 2025 Apr 2;11:129. doi: 10.1038/s41420-025-02425-9 (PMC11965325; doi:10.1038/s41420-025-02425-9)

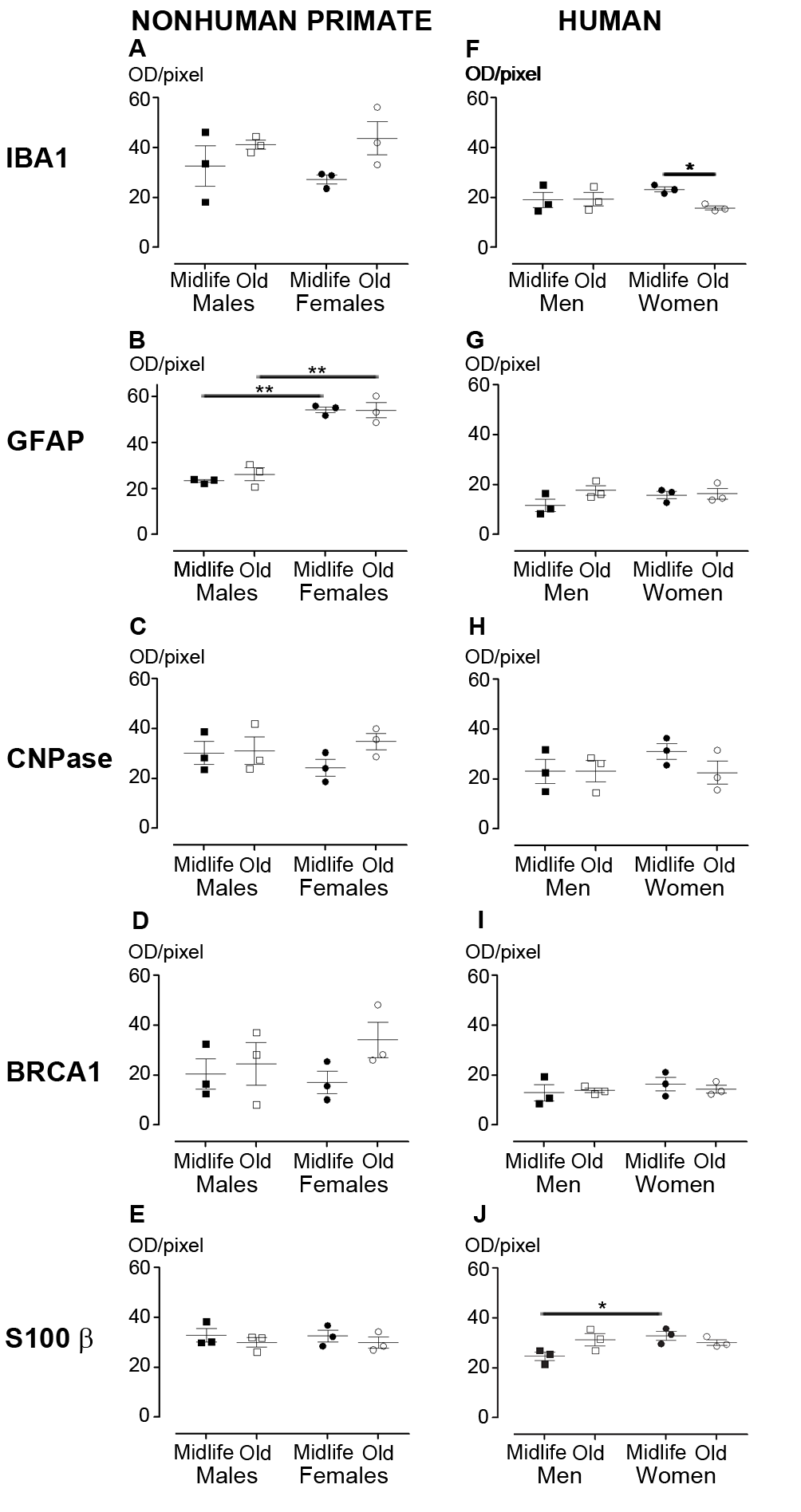

Supplement: Supplementary file 2 — Supplementary Figure 1 : Mean expression levels of all markers in the nonhuman primate and human spinal cord [file 41420_2025_2425_MOESM2_ESM.png]

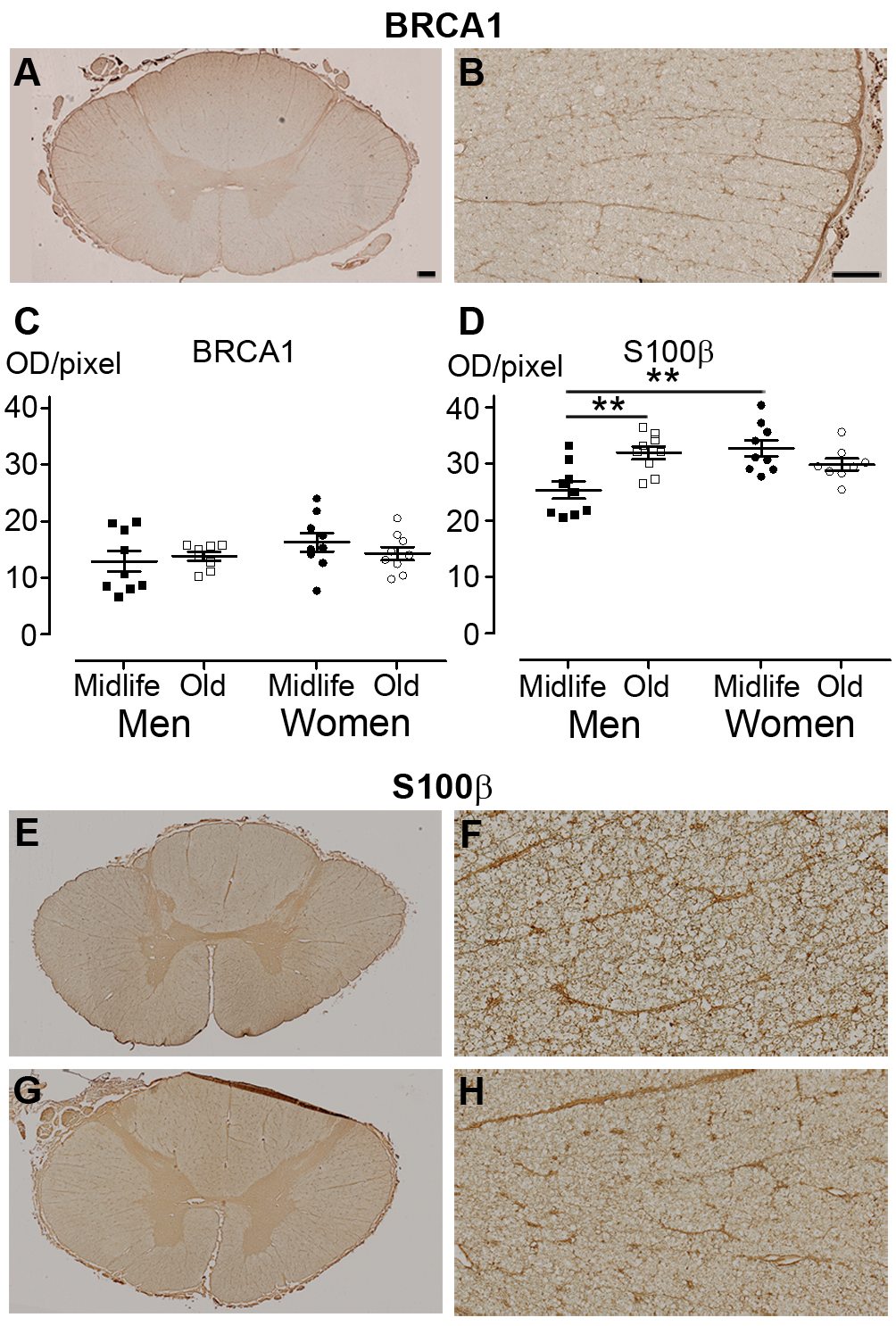

Supplement: Supplementary file 3 — Supplementary Figure 2 : BRCA1 and S100β expression in the human spinal cord to further characterize glial cells [file 41420_2025_2425_MOESM3_ESM.png]

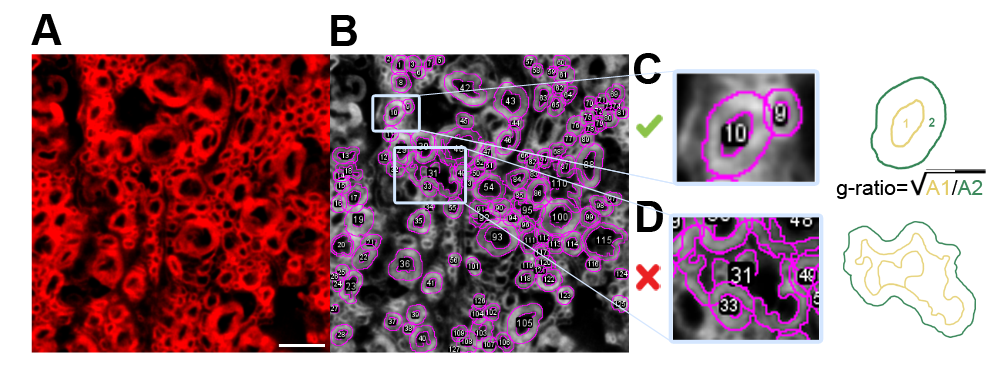

Supplement: Supplementary file 4 — Supplementary Figure 3: Method for quantifying the myelin g-ratio [file 41420_2025_2425_MOESM4_ESM.png]
